# The Growth Inhibition of Polyethylene Nanoplastics on the Bait-Microalgae *Isochrysis galbana* Based on the Transcriptome Analysis

**DOI:** 10.3390/microorganisms11051108

**Published:** 2023-04-24

**Authors:** Xinfeng Xiao, Wenfang Li, Shuangwei Li, Xingsheng Zuo, Jie Liu, Linke Guo, Xiao Lu, Linlin Zhang

**Affiliations:** College of Safety & Environmental Engineering, Shandong University of Science & Technology, Qingdao 266510, Chinazuoxs0406@163.com (X.Z.);

**Keywords:** microplastics, *Isochrysis galbana*, transcriptome analysis, bacterial community

## Abstract

The adverse effects of microplastics on microalgae species have been extensively studied, but their impact on the bait microalgae entering the food chain has not been well understood. This study investigated the cytological and physiological response of *Isochrysis galbana* to polyethylene microplastics (PE-MPs, 10 μm) and nanoplastics (PE-NPs, 50 nm). The results showed that PE-MPs had no significant impact on *I. galbana*, while PsE-NPs obviously inhibited cell growth, reduced chlorophyll content, and caused a decline in carotenoids and soluble protein. These changes in the quality of *I. galbana* could negatively affect its use as aquaculture feed. To understand the molecular response mechanism of *I. galbana* to PE-NPs, transcriptome sequencing was performed. The result revealed that the TCA cycle, purine metabolism, and some key amino acid syntheses were down-regulated by PE-NPs, while the Calvin cycle and fatty acid metabolism were up-regulated to tolerate PE-NP pressure. Microbial analysis showed that the bacterial community structure associated with *I. galbana* was significantly altered at the species level by PE-NPs. In conclusion, this study provides new insights into the physiological stress response caused by microplastic pollution based on transcriptome and bacterial community analysis. The findings highlight the need to mitigate the release of microplastics into the environment to prevent their harmful effects on aquatic ecosystems and will be helpful in understanding the impact of polyethylene nanoplastics on the bait microalgae.

## 1. Introduction

Plastic has become an indispensable part of modern society, but the improper disposal of plastic waste has resulted in numerous environmental consequences [1]. When plastic waste is discarded in the environment, it breaks down into smaller fragments, some of which are smaller than 5 mm in diameter and are refined as microplastics (MPs) [2]. MPs now have been found in freshwater, marine, sediment, and terrestrial environments [3]. The ingestion or entanglement of plastic has been found to pose a significant risk to the health of marine organisms and has become a popular research topic globally [4].

Microalgae, as primary producers and the base of the marine trophic chain, play a crucial role in maintaining the balance of marine ecosystems [5,6]. Additionally, microalgae were also associated with biodegradable, renewable, low-emission profiles, and low carbon footprints because of their outstanding photosynthesis and carbon fixation efficiency [7,8]. Recent studies showed that MPs have detrimental impacts on microalgae, including a decrease in chlorophyll content and photosynthetic efficiency, morphological changes, and the induction of oxidative stress and membrane destruction [9]. For instance, it was found that polystyrene microplastics (PS-MPs) significantly reduced the content of chlorophyll a (*Chl a*) and induced the accumulation of reactive oxygen species (ROS), resulting in oxidative damage to cells of *Euglena gracilis* [10]. *Isochrysis galbana* is one of the most commonly used marine unicellular algae in many mariculture systems because of its fast growth, balanced nutrients (protein, fatty acids, carbohydrates), and various bioactive substances [11]. Several studies have reported the negative effects of MPs on the quality and changes in carbohydrate content, as well as the food dilution effect. Changes in lipid and fatty acid composition also affect the growth, reproductive capacity, and fitness of aquatic invertebrates and fish [12]. Therefore, the impact of MPs on the bait microalgae *I. galbana* needs further exploration.

Generally, algae-associated symbiotic bacteria have a significant effect on the physiology and metabolism of algae by facilitating or inhibiting the growth of the host algae. The change in community structure was a common strategy for microbial communities to cope with pollutants. For example, the structure of the microbial community can be markedly changed either under short-term or long-term exposure to pollutants [13]. *Pseudomonas stutzeri* inhibited the growth of *I. galbana* through direct contact and enhanced algicidal effects with an increase in concentration and treatment duration. Additionally, the chlorophyll contents decreased by 23–74% compared with the axenic culture over the period of 6 days on the co-culture system [14]. However, limited information is available on the influence of MPs on the bacterial community and structure associated with *I. galbana*.

In the present study, we employed the typical polyethylene plastics with a size of 10 μm (polyethylene microplastics, PE-MPs) and 50 nm (polyethylene nanoplastics, PE-NPs), respectively, to assess their influence on the bait microalgae *I. galbana*. To further understand the physiological response mechanism of microplastic exposure, we carried out transcriptomic profiles of the microalgae and bacterial community composition analysis with 16s rRNA gene amplicon sequencing.

## 2. Materials and Methods

### 2.1. Microalgae Strain and Growth Analysis

Polyethylene microplastics (PE-MPs) and nanoparticles (PE-NPs) were both procured from Zhongxin Plastics Co., Ltd., Dongguan, China. Deionized water was used as the medium for preparing the plastic mixtures, and all solutions were sonicated at 120 W for 30 min before dilution. The microalgae *I*. *galbana* was obtained from the Institute of Oceanography, Chinese Academy of Sciences. Seawater was filtered through 0.45-μm cellulose acetate membranes and then sterilized by autoclaving. *I. galbana* was cultured in the f/2 medium supplemented with silicate, under conditions of constant light intensity (4000 Lux) at 23 ± 1 °C [15]. Once the microalgae reached the early exponential growth phase, PE-MPs and PE-NPs were separately added into the sterile medium and were denoted as the MPs-treatment group. A group without any plastic treatment under the same conditions served as the control group. The microalgal cell density was tested every 24 h for 14 days, and all experiments were performed in triplicate.

### 2.2. Determination of Microalgae Biomass, Pigment, and Soluble Protein Content

Microalgae biomass was analyzed through optical density measurements at 680 nm using a spectrophotometer. To ensure accuracy, control groups were established for each concentration to prevent the influence of plastic particles on algal absorption. The determination of *Chl a* and total carotenoid concentration was conducted using a spectrophotometric method, as described in the literature [16]. Specifically, *I. galbana* cells were collected and centrifuged at 8000× *g* for 5 min, and then mixed with 99.8% (*v*/*v*) methanol. After the microalgae cells were immersed in the centrifugal tube at 4 °C for 24 h, the absorbance was read at 470, 653, and 666 nm using a microplate reader. To determine the soluble protein content, cells treated with or without MPs were resuspended in PBS (phosphate buffered saline, pH 7.8) and harvested using centrifugation at 5000× *g* for 10 min. The Bradford method was used to determine the soluble protein content. Specifically, a solution of 10.0 mL phosphate buffer (pH 7.8) was mixed with the same volume of algae solution, followed by sonication for 30 min. The supernatant was then collected by centrifugation at 8000× *g* for 5 min at 4 °C to measure the soluble protein content.

### 2.3. Measurement of MDA Levels and Antioxidant Enzyme Activity

To investigate whether excessive ROS was induced by PE-NPs, an indicator of lipid peroxidation of malondialdehyde (MDA) was measured according to a previous study [15]. Microalgal cells were collected as described above, and 0.3 mL of supernatant was added to a solution containing 3 mL of 20% TCA (including 1% thiobarbituric acid). The mixed solution was heated to 90 °C and quickly cooled. After centrifugation for 10 min, the supernatant’s absorbance was measured at 532 nm. Preparation of antioxidant enzyme extracts treated with PE-NPs and control was carried out, and the obtained supernatants were used for further analysis. Superoxide dismutase (SOD) activity was measured in fresh extracts by monitoring the absorbance change of nitro blue tetrazolium (NBT), as described previously [16]. Peroxidase (POX) activity was assayed by measuring the absorbance change due to H_2_O_2_ consumption under the assay conditions of 25 °C.

### 2.4. Transcriptome Sequencing and Analysis

To gain new insights into the physiological response and metabolic processes, *I. galbana* cells were analyzed after a 14-day exposure to 50 mg/L of PE-NPs. Microalgae cells treated and untreated with PE-NPs were collected, respectively, and total RNA was extracted and then used for transcriptome sequencing. Library construction and sequencing were carried out by Berry Genomics Corporation (Beijing, China). Gene Ontology (GO) and Kyoto Encyclopedia of Genes and Genomes (KEGG) enrichment analysis based on the differentially expressed genes (DEGs) were performed following literature descriptions [17]. For biological replicates, genes with *p*-values less than 0.05 and simultaneously met the conditions of fold changes of 1.5 or 0.66 were identified as differentially expressed genes (DEGs) in response to PE-NPs exposure. Pathway enrichment analysis of DEGs based on the KEGG database was carried out. The software HemI (Heatmap Illustrator, version 1.0., Beijing, China) was used for further analysis of DEGs.

### 2.5. Analysis of Microbial Community Structure and Diversity

Genomic DNA was extracted from bacterial cultures at the end of a 14-day growth experiment, using a previously described high-quality extraction method [18]. The V3–V4 regions of bacterial 16S rRNA genes were amplified using specific primers with unique barcodes, and operational taxonomic units (OTUs) were obtained under a 97% identity threshold using UCLUST. Paired-end sequencing was performed on an Illumina Hiseq platform (Novogene Co., Ltd., Beijing, China) according to a standard protocol.

### 2.6. Co-Culture Experiments

Microalgal cultures were collected and spread on 2216E agar plates with various dilutions. After two days of cultivation, a single colony was incubated with 2216E broth to sequence the 16S rRNA gene, which was amplified by PCR using the primers 27F and 1541R. The resulting sequences were compared to reference sequences using NCBI-BLAST and phylogenetic analysis was conducted using MEGA-X software (version 7.0) [19]. To further investigate the interactions between microalgae and bacteria, co-culture experiments were conducted. A single colony of bacteria was plated freshly on 2216E agar plates and inoculated into 2216E broth for 24 h in a shaking incubator (28 °C, 180 rpm). When axenic microalgae reached the exponential phase of growth, each bacterial strain was added to the algal culture at a concentration of 1% (*v*/*v*). Each sample was counted three times to obtain the average cell density, which was determined by counting cells with a hemocytometer (XB-K-25, Xiangbo Co., Ltd., Changsha, China) under a light microscope.

### 2.7. Statistical Analysis

All data obtained during the study are given as the mean ± standard deviation (SD). The statistical analyses were completed by using IBM SPSS 22 version (IBM Co., Ltd., Armonk, NY, USA), and graphics rendering was carried out using Graphpad Prism 8.0 (GraphPad Software, San Diego, CA, USA). The differences between samples are identified as significant when the *p*-value is <0.05.

## 3. Results

### 3.1. Microalgal Growth and Physiology Analysis

**Figure 1:**
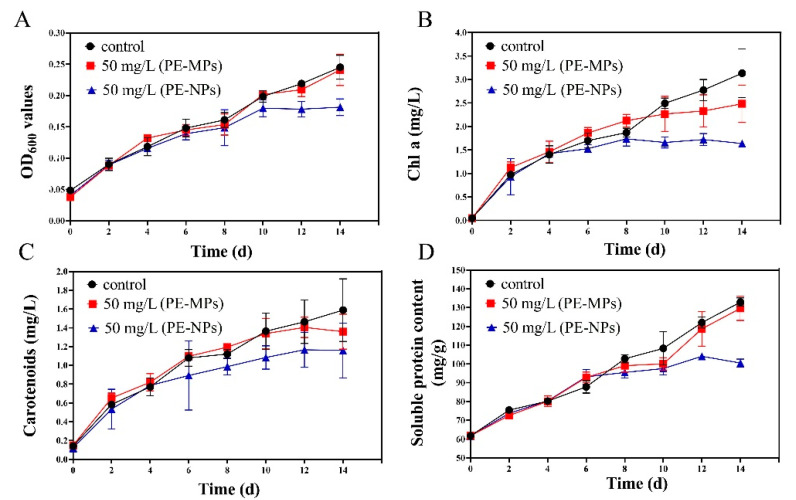
Physiological responses of microalgae of *I. galbana* to different particle size MPs at the concentration of 50 mg/L. The effect of PE-MPs and PE-NPs on the growth (**A**), *Chl a* content (**B**), carotenoids content (**C**), and soluble protein content (**D**).

Figure 1A showed that no significant difference in the microalgal growth was observed when PE-MPs (50 mg/L) were present in the culture. However, the cell growth was obviously inhibited by PE-NPs (50 mg/L) compared to the control group and the growth inhibition rate of the PE-NPs was 26.12% on the 14th day. The observed changes in *Chl a* and carotenoid content were consistent with the growth curve of *I. galbana* exposed to the MPs pollutant (Figure 1B,C). Although the presence of PE-MPs did not result in a significant decrease in *Chl a* and carotenoid content, the content of these pigments decreased by 47.6% and 27.0%, respectively, compared to the control group when *I. galbana* was exposed to PE-NPs over the 14-day period. This result indicated that PE-NPs induced a more marked decrease in *Chl a* and carotenoid content than PE-MPs did. For the soluble protein content (Figure 1D), it can be found that PE-NPs resulted in a 24.40% reduction in soluble protein content compared to the control group after a 14-day culture, while PE-MPs did not have a significant effect on *I. galbana*.

### 3.2. Test of Antioxidant Enzymes Activity

**Figure 2:**
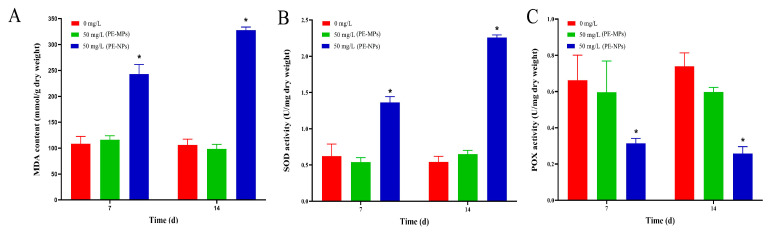
Effects of PE-MPs and PE-NPs on the *I. galbana.* (**A**) MDA content, (**B**) SOD enzymatic activity; (**C**) POX enzymatic activity. Significant differences between treatments for PE microplastics were noted with an asterisk (* *p* < 0.05). The error bars were standard error (*n* = 3).

The results presented in Figure 2A indicated that there was no significant change in MDA content between the control and PE-MPs-treatment groups over the course of 14 days. However, the PE-NPs treatment group exhibited a higher MDA content, with a 2.24-fold and 3.09-fold increase compared to the control group at 7 and 14 days, respectively. This result suggested that the plastic nanoparticles induced excess ROS and lipid peroxidation levels. Additionally, SOD activity, an important ROS-scavenging enzyme in microalgae, exhibited a similar trend to the MDA content. On exposure to PE-NPs, SOD activity increased by 4.94-fold and 4.15-fold at 7 and 14 days, respectively (Figure 2B). In contrast, the changing trend of POX activity was completely different from that of SOD (Figure 2C). POX was significantly lower in the PE-NPs treatment group compared to the control group, with a decline rate of 65.0% at 14 days.

### 3.3. Overall Transcriptional Response Profile of I. galbana to MPs Exposure

We conducted GO functional enrichment analysis on the DEGs and categorized the GO terms into three groups: molecular function (MF), cell component (CC), and biological process (BP), with a total of 22 subcategories (Figure S1). Additionally, we mapped the DEGs to terms in the KEGG database (Figure S2) and assigned 1248 genes to 20 KEGG pathways. The top five significantly enriched pathways were ribosome, spliceosome, protein processing in the endoplasmic reticulum, RNA transport, and ribosome biogenesis in eukaryotes. Furthermore, the Eukaryotic Orthologous Group (KOG) functional classification was shown in Figure S3. These findings provide valuable insights into the molecular mechanisms underlying the response of *I. galbana* to plastic nanoparticle exposure and may have broader implications for understanding the impacts of plastic pollution on aquatic ecosystems.

### 3.4. Analysis of DEGs Related to Cell Metabolism and Photosynthesis

**Figure 3:**
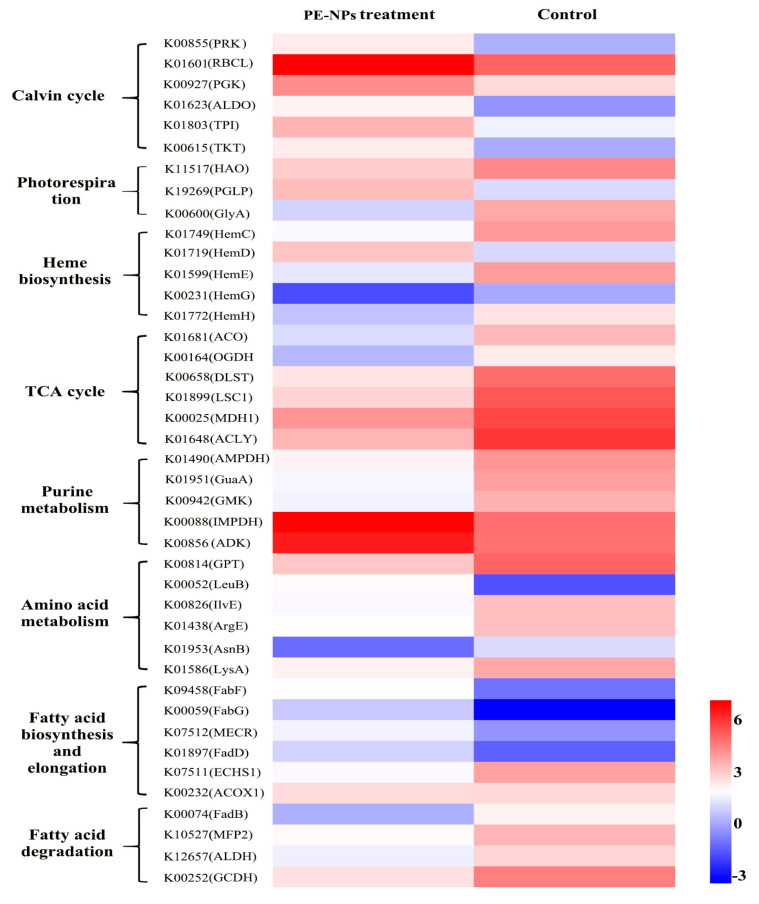
Transcriptome, clustering, and heatmap analysis of DEGs. The relative gene expression levels (fold change > 1.5 or fold change < 0.67, and *p* < 0.05) were clustered and analyzed. Red represented up-regulation and blue represented down-regulation.

In order to gain insight into the mechanisms involved in the effect of PE-NPs on the metabolism and photosynthesis of *I. galbana*, we analyzed a full set of DEGs (Figure 3).

**Calvin cycle.** Calvin cycle is the primary pathway for fixing CO_2_ and provides initial organic compounds and energy for subsequent metabolism. Upon exposure to PE-NPs, most of the DEGs related to the Calvin cycle were up-regulated, including PRK (phosphoribulokinase), RBCL (ribulose-bisphosphate carboxylase), PGK (phosphoglycerate kinase), GAPA (glyceraldehyde 3-phosphate dehydrogenase), ALDO (fructose-bisphosphate aldolase, class I), TPI (triosephosphate isomerase), and TKT (transketolase). PRK is a key nuclear-encoded plastid-localized enzyme unique to the Calvin cycle. As the catalytic large subunit of ribulose-1,5-bisphosphate carboxylase-oxygenase (RuBisCO), RBCL is considered the rate-limiting enzyme for CO_2_ fixation in the Calvin cycle and is associated with lipid accumulation. GAPA and ALDO also act as essential enzymes in the Calvin cycle. Thus, the increased activity of the Calvin cycle pathway helps *I. galbana* maintain its CO_2_ fixation rate and accumulate more energy and metabolites under unfavorable conditions.

**Photorespiration.** Photorespiration is a costly respiratory pathway that requires a high amount of energy and utilizes 2-phosphoglycolate to release CO_2_ while simultaneously taking up oxygen in a light-dependent manner [20]. In Figure 3, DEGs involved in photorespiration, including PGLP (phosphoglycolate phosphatase), HAO ((S)-2-hydroxy-acid oxidase), and GlyA (glycine hydroxymethyltransferase), were identified. PGLP converts 2-phosphoglycolate to glycolate, which is then converted to glycine. Glycine is proposed to be exported to the mitochondrion and converted to serine. The down-regulation of PGLP, HAO, and GlyA suggests that the photorespiration process is inhibited, which conserves energy consumption for *I. galbana* under PE-NPs stress. However, GlyA is responsible for the metabolism of glycine, which is a precursor for heme and *Chl a*. The down-regulation of GlyA impedes the production of glycine and is unfavorable for *Chl a* biosynthesis.

**Heme biosynthesis pathway.** Heme is a critical component of many groups of proteins that includes cytochromes (for mitochondrial respiratory chain electron transfer), oxidases and peroxidases, catalases, and synthases [21]. The transcriptome analysis revealed that 13 genes are involved in the heme biosynthesis pathway (M00121), including hemC, hemD, hemF, hemG, hemH, and hemL, all of which were significantly down-regulated. These genes encode enzymes that catalyze the conversion of uroporphyrinogen III to coproporphyrinogen III, protoporphyrinogen IX, and finally to protoporphyrin IX, which is a crucial precursor for chlorophyll biosynthesis. Therefore, the changes in expression levels of these genes were found to be associated with the synthesis of *Chl a*, and to some extent, accounted for the decline in *Chl a*. Additionally, ROS production is linked to the photosynthetic electron transport in chloroplasts as well as the oxygen storage and transport molecules, myoglobin and hemoglobin. As downstream metabolites of the photosynthetic pathway, CAT, POX, and cytochrome c can act as antioxidant molecules to scavenge ROS production. Hence, the down-regulation of genes involved in photosynthesis may have adverse effects on ROS elimination, leading to ROS accumulation.

**Purine metabolism.** Exposure to PE-NPs resulted in a decrease in the expression of genes related to purine metabolism, including IMPDH (IMP dehydrogenase), GuaA (GMP synthase), GMK (guanylate kinase), AMPD (AMP deaminase), and ADK (adenylate kinase). The down-regulation of IMPDH and AMPD in purine nucleotide metabolism could inhibit the formation of XMP (Xanthosine monophosphate) and AMP (Adenosine monophosphate), respectively, which indicates a disturbance in purine metabolism and a decline in DNA replication. 

**Tricarboxylic acid (TCA) cycle.** Exposure to PE-MPs consistently inhibited the DEGs coding nearly all the key enzymes in the TCA cycle, including ACO (aconitate hydratase), OGDH (oxoglutarate dehydrogenase), IDH3 (isocitrate dehydrogenase), DLST (dihydrolipoamide succinyltransferase), LSC1 (succinyl-CoA synthetase alpha subunit), MDH1 (malate dehydrogenase), and ACLY (ATP citrate (pro-S)-lyase) (Figure 3). Notably, the genes coding OGDH and DLST were down-regulated by 74.0% and 81.2%, respectively, under PE-NP exposure to stress. As the TCA cycle is a canonical energy pathway, the down-regulation of these DEGs may have adverse effects on amino acid synthesis, nitrogen metabolism, and acetyl-CoA. Similar results were observed in the cells of *Synechocystis* sp. exposed to NPs/MPs, as the hetero aggregation between NPs/MPs and microalgae cells could hinder the transfer of energy and substances, thus blocking carbon fixation in photosynthesis and subsequent carbohydrate synthesis.

**Figure 4:**
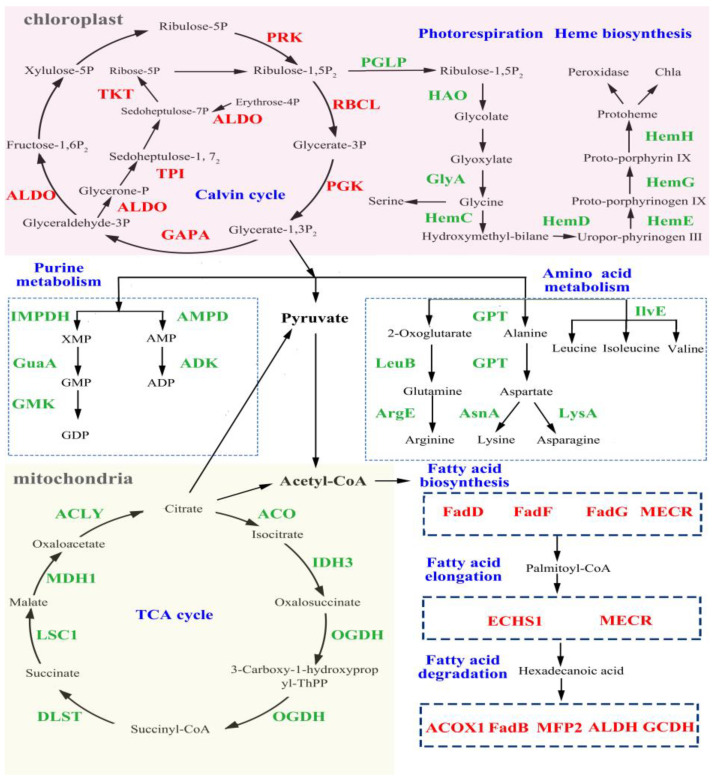
Cellular metabolism pathways of *I. galbana* exposed to PE-MPs, the color scale represents the down-regulation (green) and up-regulation (red) of genes.

**Amino acid metabolism.** Further transcriptomic sequencing analysis revealed that the expression levels of DEGs related to amino acids were altered compared to the control (Figure 3 and Figure 4). In the biosynthesis pathway of amino acids, down-regulated genes included GPT (alanine transaminase), AsnA (aspartate--ammonia ligase), IlvE (branched-chain amino acid aminotransferase), LysA (diaminopimelate decarboxylase), and ArgE (acetylornithine deacetylase). The significantly changed genes were related to alanine, leucine, isoleucine, valine, lysine, arginine, glutamine, and asparagine. Glutamine is the most abundant amino acid in many organisms and is usually regarded as a major nitrogen donor in the biosynthesis of many organic N compounds, such as purines, pyrimidines, and other amino acids. Additionally, alanine, leucine, and lysine are metabolized as carbon skeletons to form acetyl-CoA. Therefore, the down-regulation of these genes could negatively regulate their synthesis or metabolism. 

**Fatty Acid Metabolism.** Fatty acids are a crucial energy source that can be degraded into acetyl-CoA to participate in the tricarboxylic acid cycle (TAC). PE-NPs affected fatty acid biosynthesis through the up-regulation of DEGs, such as FabG (3-ketoacyl-ACP reductase), FabF (3-oxoacyl-ACP synthase II), FabD (malonyl-CoA:ACP transacylase), and MECR (mitochondrial enoyl-[acyl-carrier protein] reductase) (Figure 3 and Figure 4). These genes were critical in fatty acid biosynthesis and elongation. The gene encoding MECR, which catalyzes the acyl-CoA with a chain length from C6 to C16, was up-regulated by 3.93-fold. FabF and FabG, which were responsible for elongation, also increased by 7.57- and 17.68-fold, respectively, compared to the control group after PE-NPs exposure. Additionally, genes related to fatty acid degradation were also significantly up-regulated, including ACOX1 (acyl-CoA oxidase), MFP2 (enoyl-CoA hydratase), FadB (enoyl-CoA hydratase), and ALDH (aldehyde dehydrogenase (NAD+)). Therefore, fatty acids might be consumed to provide more energy for *I. galbana* to adapt to the PE-NPs stress.

### 3.5. Analysis of DEGs Related to Antioxidant Defense

**Figure 5:**
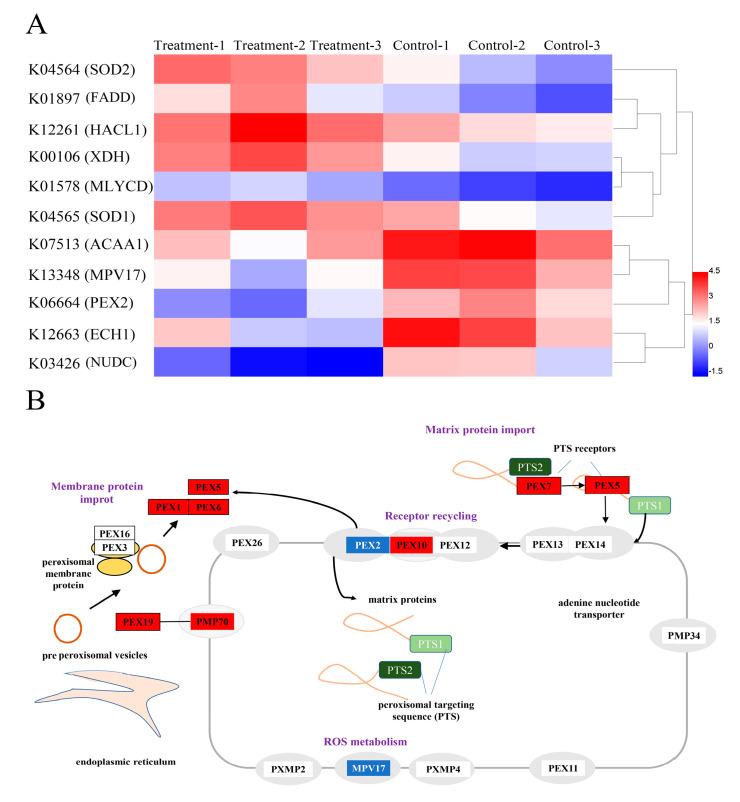
Transcriptome, clustering, and heatmap analyses of DEGs involved in ROS production and scavenging. (**A**) the results of these DEGs are presented as a heat map. Red represented up-regulation and blue represented down-regulation. (**B**) the enriched DEGs were mapped to the Peroxisome. ‘Blue’ blocks represent down-regulation while ‘red’ blocks represent up-regulation.

Figure 5 showed that a total of 11 DEGs related to antioxidant defense were identified, including SOD1, SOD2, FADD, HACL1, XDH, ACAA1, MPV17, PEX2, ECH1, and NUDC (Figure 5A). As ROS-scavenging enzyme subunits, both SOD1 and SOD2 had a remarkable up-regulation, which was consistent with the change in SOD enzymatic activity in this study. Moreover, eight up-regulated DEGs, including PEX1, PEX2, PEX5, PEX6, PEX7, PEX10, PEX19, and PEX70, were enriched in the Peroxisome (ko04146). Peroxisomes are single-membrane-bound organelles that have the function of degrading fatty acids (β-oxidation) and detoxifying ROS by diverse enzymatic and non-enzymatic antioxidants, such as SOD, catalase (CAT), ascorbate peroxidase (APX), reduced glutathione (GSH), and ascorbic acid (Figure 5B). Thus, these up-regulated DEGs may enhance the ability of the peroxisomal ROS-scavenging system, resulting in an increase in the antioxidant enzyme SOD activity to remove excess ROS. However, excess ROS triggered by environmental stress can also lead to the oxidative impairment of peroxisomal proteins and peroxisomal matrix proteins, further aggravating ROS generation. Therefore, the interaction between the change of DEGs in the peroxisomes and ROS induced by MPs needs to be further explored in the future. Additionally, the expression level of MPV17 was significantly down-regulated by PE-NPs. MPV17 is a mitochondrial inner membrane protein with four hydrophobic regions, and it allows small molecules to pass through the membrane. It has been reported that PyMPV17 in Chlamydomonas could enhance osmotic stress tolerance by reducing MDA production resulting from oxidative damage under stress conditions. Thus, it can be concluded that the down-regulation of the transcription level of the gene coding for MPV17 attenuated the ROS-scavenging ability of microalgae.

### 3.6. Bacterial Community Composition Analysis

**Figure 6:**
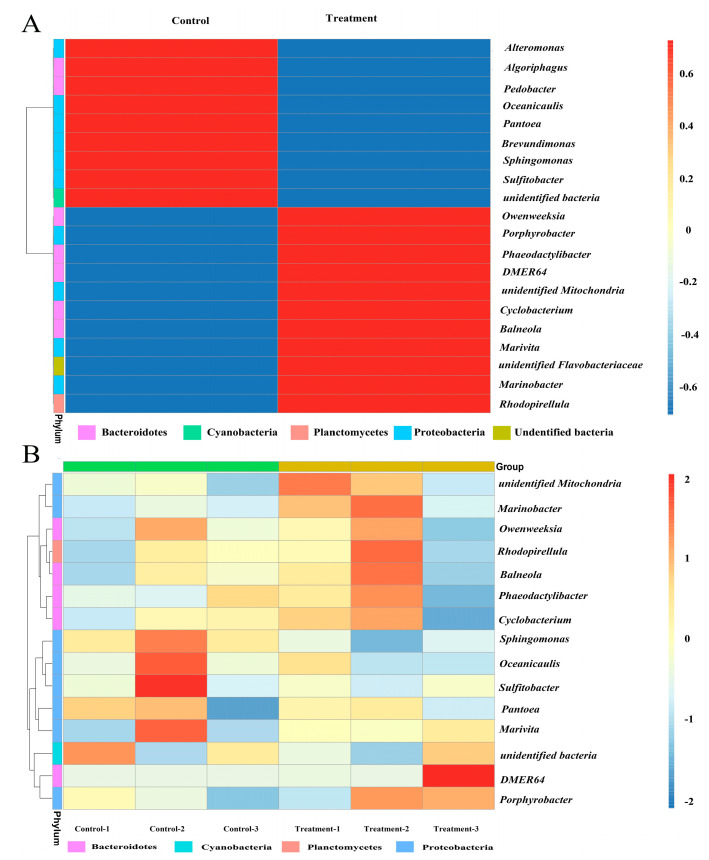
Heat map analysis of bacterial community between control and PE-NPs treatment groups. (**A**) relative abundances of the top-20 abundant groups within different communities are shown at phylum level. (**B**) relative abundances of the top-15 abundant groups within different communities are shown at genus level. Sequences that cannot be classified into the known group and other smaller phyla (genus) are assigned as “undentified”.

There is increasing evidence that specific symbiotic bacteria have a function in the growth of *I. galbana*, and their presence often affects the growth of the microalgae [20]. To further analyze the growth inhibition mechanism of plastics on microalgae, we characterized the communities and microbial diversity of bacteria associated with *I. galbana* based on 16S rDNA amplicon Illumina sequencing. In the control group, Proteobacteria showed the highest average relative abundance (46.66%), followed by Cyanobacteria with 30.35% (Figure S4A). After PE-NPs treatment, Proteobacteria still had the highest average relative abundance of 51.00%, but the relative abundance of Cyanobacteria decreased from 30.35% to 22.78%, and the abundance of Bacteroides increased from 19.31% to 22.82% (Figure S4A). It is evident that the relative abundance of bacteria affiliated with Proteobacteria was the most abundant phylum in different groups, consistent with previous observations [16]. Further examination showed that bacterial community diversity was significantly changed at the species level (Figure S4B). The relative abundance of bacteria, such as *Alteromonas*, *Algoriphagus*, *Pantoea*, *Pedobacter*, *Sphingomonas*, and others, accounted for a predominant proportion in the control group but declined on day 14 (Figure 6A,B). Simultaneously, there were significant increases in the relative abundances of bacteria, including *Marinobacter*, *Owenweeksia*, *Phaeodactylibacter*, *Porphyrobacter*, *Balneola*, *Phaeodactylibacter*, and *Rhodopirellula*, in the PE-NPs treatment group compared to the control group.

### 3.7. The Effect of Symbiotic Bacteria on the Microalgal Cell Growth

**Figure 7:**
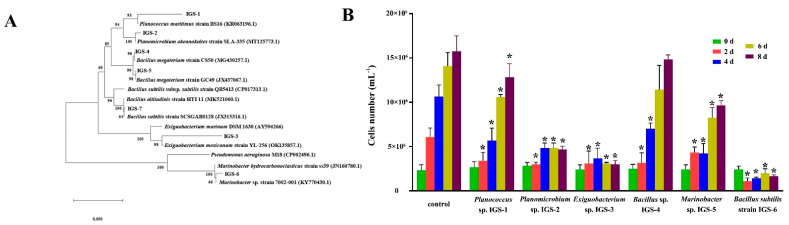
The effect of symbiosis bacteria on the microalgal cell growth. (**A**) Phylogenetic tree analysis based on 16S rDNA sequences of the symbiotic cultivable bacteria strains isolated from *I. galbana* culture. (**B**) The influence of symbiotic bacteria strains on the *I. galbana* growth in the co-culture experiment, (* *p* < 0.05).

In this study, a total of six different species of bacteria were isolated from the microalgal culture and named IGS-1, IGS-2, IGS-3, IGS-4, IGS-5, and IGS-6, respectively. Based on 16S rRNA gene sequence alignment and phylogenetic tree analysis (see Figure 7A), these algae-associated bacterial strains were identified as *Planococcus* sp. IGS-1, *Planomicrobium* sp. IGS-2, *Exiguobacterium* sp. IGS-3, *Bacillus* sp. IGS-4, *Marinobacter* sp. IGS-5, and *Bacillus* subtilis strain IGS-6. To the best of our knowledge, this is the first report regarding the isolation and characterization of the *Planococcus* and *Planomicrobium* species from the microalgae culture broth. It is worth noting that the relative abundance of *Marinobacter* sp. in the PE-NPs treatment group was higher than in the control. The increasing cell concentration of the bacterium *Marinobacter* sp. would negatively affect microalgal growth. As shown in Figure 7B, most of the algae-associated bacterial strains had an obvious adverse effect on the growth of *I. galbana* except *Bacillus* sp. IGS-4 after an 8-day co-culture. *Bacillus* subtilis strain IGS-6 exhibited the highest growth inhibition rate of 89.42% on microalgal cells. Herein, it can be found that the growth inhibition ratio was 18.52% when *Planococcus* sp. IGS-1 was present in the microalgal culture. In addition, *Planomicrobium* sp. IGS-2, *Exiguobacterium* sp. IGS-3, and *Marinobacter* sp. IGS-5 had growth inhibition ratios of 70.37%, 81.95%, and 38.62%, respectively.

## 4. Discussion

**Figure 8:**
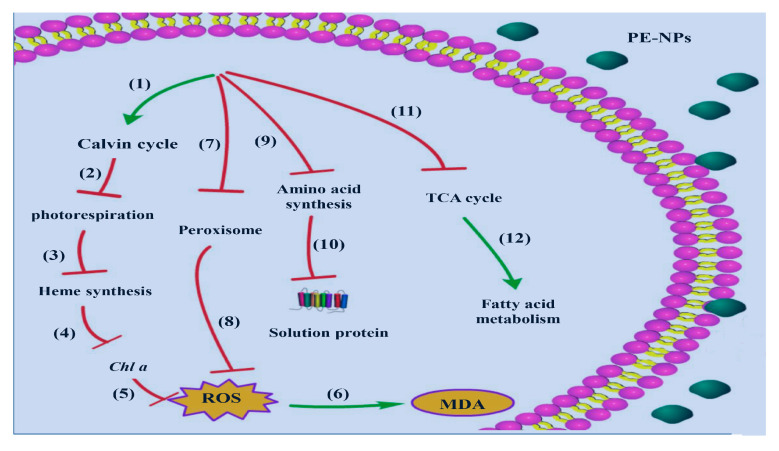
The proposed model of PE-NPs action on *I. galbana* cells. PE-NPs exposure led to the expression level up-regulation of genes in the Calvin cycle (1). The down-regulation of photorespiration (2) and Heme synthesis (3) contributed to *Chl a* content reduction (4) and ROS accumulation (5). Excess ROS finally damaged the intracellular macromolecules and gave rise to MDA content increase through oxidation reaction (6). The gene expression level change in the Peroxisome (7) resulting from PE-NPs pollution partly caused the ROS increase (8). The genes involved in the biosynthesis of amino acids were altered (9), which lead to the decline of protein content (10) and influenced the feed quality. PE-NPs also inhibited the TCA cycle (11). As a response to the adverse environment, fatty acid metabolism was up-regulated (12).

This study demonstrated that PE-MPs did not produce an obvious effect on microalgae growth, but PE-NPs exhibited obvious toxicity towards *I. galbana*, including growth inhibition as well as a reduction in *Chl a*, carotenoid, and protein content (Figure 8). The interactions between MPs and microalgae may vary with microalgal species and plastic materials, and the size of MPs was of paramount importance for their effect on organisms [22]. Smaller MPs may have stronger growth inhibition than larger ones. Notably, 50 nm PS-NPs exhibited a significant inhibition effect on the growth of *Dunaliella tertiolecta*, while 6 μm PS-MPs showed no significant influence on microalgae [23]. Similar results were also found in the *E. gracilis* when it was exposed to PS microbeads with diameters of 5 μm and 100 nm [8]. Smaller particles may generate the growth inhibition effect on the microalgae by blocking algal pores or gas exchanges and entering cells through cell wall pores [24].

The negative impact of PE-NPs on the pigment content of microalgae observed in our study is consistent with findings in other marine microalgae, such as *Scenedesmus obliquus*, *Tetraselmis chuii*, *Skeletonema costatum*, and *Dunaliella salina* [25,26]. The detrimental effect of smaller plastic particles on microalgae is not limited to a single species or type of plastic. The larger specific surface area of smaller MPs may play a role in hindering the natural flow of energy and substances in microalgae, leading to damage and decreased pigment content. Furthermore, the decrease in chlorophyll content may be due to a decrease in the expression of photosynthesis genes, as well as surface adsorption of MPs leading to an increase in energy demand, electron accumulation, and excess production of reactive oxygen species (ROS). As the structural and functional components of the antioxidant systems, carotenoids quench ROS by reacting with lipid peroxidation products and protect chlorophylls, lipids, proteins, and DNA from oxidative damage. In addition, carotenoids could be used as an important component of feed for aquaculture and produced by microalgae to large levels after exposure to specific environmental stimuli. Thus, the content decline of carotenoids may exert a negative effect on photosynthesis and ROS scavenging, environmental adaptation, and the food quality of crucial resources at the base of aquatic food webs [27]. Previous studies have shown that plastic particles have the potential to affect microalgae lipid, fatty acid composition, and carbohydrate and protein metabolism. For example, a decrease in protein yield was observed in the microalgae *Acutodesmus obliquus* with increasing concentrations of HDPE, PP, and PVC. This decrease in protein concentration may be attributed to an adaptation mechanism to cope with stress at higher concentrations of plastic particles [28]. 

The overwhelming production of ROS induced by PE-NPs in *I. galbana* are consistent with previous studies on other microalgae, including *Chlorella pyrenoidosa*, *E. gracilis*, and *Chlorella* sp. L38 [29,30,31]. Excessive ROS can damage the structure of DNA, carbohydrates, lipids, proteins, and other cellular macromolecules once the intrinsic antioxidant defenses of cells are overloaded. In response to oxidative stress, ROS-scavenging enzymes such as SOD, POD, and CAT usually exhibit a significant increase to reduce the ROS level in microalgae. For example, the SOD activity of *Cladocopium goreaui* significantly increased at 1 day of exposure to polystyrene MPs compared to the control group [32]. SOD and CAT activities of *C. pyrenoidosa* also significantly increased with an increase in PE1000 and PA1000 content from 10 mg/L to 100 mg/L after 96 h of exposure [33]. Our results showed that large amounts of MDA were produced under PE-NPs stress, and SOD activity increased to cope with oxidative damage in *I. galbana*. However, PE-NPs repressed the POX activity and weakened the ability of *I. galbana* to prevent the removal of ROS, further promoting the accumulation of ROS and leading to a reduction in the detoxification response of *I. galbana* (Figure 8).

Bacterial communities commonly associated with algal stock cultures are often dominated by Proteobacteria and Bacteroides. Ling et al. found that the relative abundance of bacteria affiliated with Alphaproteobacteria and Gammaproteobacteria accounted for a predominant proportion of seven bait microalgae [18]. Dominant bacteria are often closely linked to the growth of algae, with bacteria affiliated with Proteobacteria, such as *Phaeodactylum tricornutum*, *Thalassiosira*. *Pseudonana*, *P. stutzeri*, and *Marinobacter sp*., significantly affecting the growth of microalgae [34]. For some microalgae, dominant bacteria may have a positive effect on their growth, such as *Marinobacter* sp., *Algoriphagus* sp., *Dinoroseobacter* sp., and *Oceanicaulis* sp. Co-cultures of *I. galbana* 3011 grown with *Algoriphagus* sp. affiliated with Bacteroidetes yielded higher chlorophyll contents than those of the axenic culture, improving microalgae growth [18]. However, other dominant bacterial strains inhibited the growth of algae by competing with algae for essential nutrients or secreting algicidal compounds. *Microbacterium* sp. competed with algae for essential nutrients, slowing down the growth rate of *Chlorella vulgaris* OW-01, and *Exophiala* sp. resulted in growth inhibition of the algae with an algicidal pattern [35]. Our results indicated that the dominant bacterial strains have been changed by PE-NPs at the species level, which is helpful in finding connections between PE-NPs and the symbiotic bacterial community. This result provides a new perspective for further unveiling the inhibition mechanism of PE-NPs to microalgae. 

*Planococcus* sp. has been isolated from diverse marine environments, but its influence on microalgae has not yet been investigated. The genus *Planomicrobium* sp. is closely related to the *Bacillus cereus* group and exhibits algicidal activity to *Gymnodinium catenatum*. In a previous study, the cell growth of the *Chlorella-Exiguobacterium* consortium was higher than those of the axenic *Chlorella* system and pure bacterial system. The co-cultured bacteria might secrete some metabolites to promote the growth of microalgae and bacteria. Ling et al. found that co-cultures of *I. galbana* 3009, *I. galbana* 3010, and *I. galbana* 3011 grown with *Marinobacter* sp. all yielded higher chlorophyll contents than those of the axenic culture [18], which is opposite to our assay results. Hence, the detailed mechanism of how *Marinobacter* sp. influences the growth of microalgae requires further investigation. As a result, we can conclude that bacteria associated with microalgae play an important role in the growth of microalgae. Additionally, the dominant bacterial strains changed by PE-NPs at the genus level can help to find connections between PE-NPs and the symbiotic bacterial community, providing a new perspective for further understanding the inhibition mechanism of PE-NPs on microalgae.

Overall, the transcriptome and biochemical analyses in this study suggest that PE-NPs affect carbohydrate metabolism pathways and symbiotic bacteria structure and composition, leading to a reduction in pigment content, high ROS accumulation, and growth inhibition. These findings reveal detailed mechanisms involved in the toxicity and adaptation of PE-MPs in *I. galbana* species.

## Data Availability

Not applicable.

## Supplementary Materials

The following supporting information can be downloaded at https://www.mdpi.com/article/10.3390/microorganisms11051108/s1, Figure S1: GO analyses of unigenes identified in *I. galbana*; Figure S2: KEGG enrichment analysis in *I. galbana* exposed to MPs compared with the control; Figure S3: Eukaryotic Orthologous Group (KOG) functional classification; Figure S4: Relative abundance of the dominant bacteria of in different groups at the phylum levels.
