# Supplementary material for: The Growth Inhibition of Polyethylene Nanoplastics on the Bait-Microalgae Isochrysis galbana Based on the Transcriptome Analysis
Source: Microorganisms. 2023 Apr 24;11(5):1108. doi: 10.3390/microorganisms11051108 (PMC10224565; doi:10.3390/microorganisms11051108)
Supplement: Supplementary file 1 [file microorganisms-11-01108-s001.zip › microorganisms-2360290-supplementary.pdf]

The Growth Inhibition of Polyethylene Nanoplastics on the Bait-Microalgae *Isochrysis galbana* Based on the Transcriptome Analysis

Xinfeng Xiao, Wenfang Li, Shuangwei Li, Xingsheng Zuo, Jie Liu, Linke Guo, Xiao Lu and Linlin Zhang *

College of Safety & Environmental Engineering, Shandong University of Science & Technology, Qingdao 266510, China; xf.xiao@163.com (X.X.); 17854205137@163.com (W.L.); lsw2798745715@163.com (S.L.); zuoxs0406@163.com (X.Z.); 17852581091@163.com (J.L.); glk163163@163.com (L.G.); luxury990103@163.com (X.L.)

* Correspondence: skd993314@sdust.edu.cn

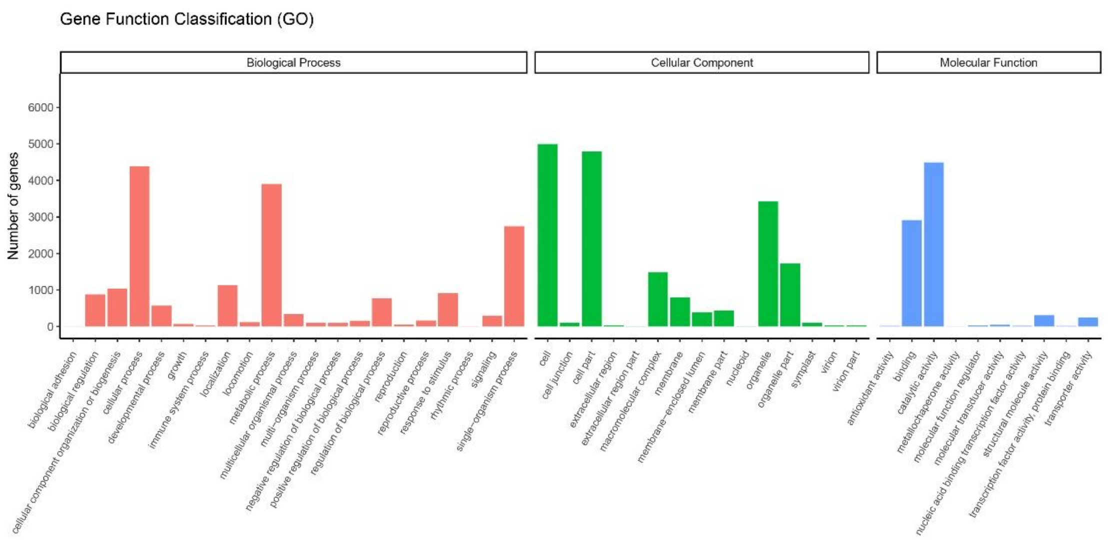

Figure S1. GO analyses of unigenes identified in *I. galbana*. These unigenes were categorized into three main GO groups (biological process, cellular component, and molecular function).

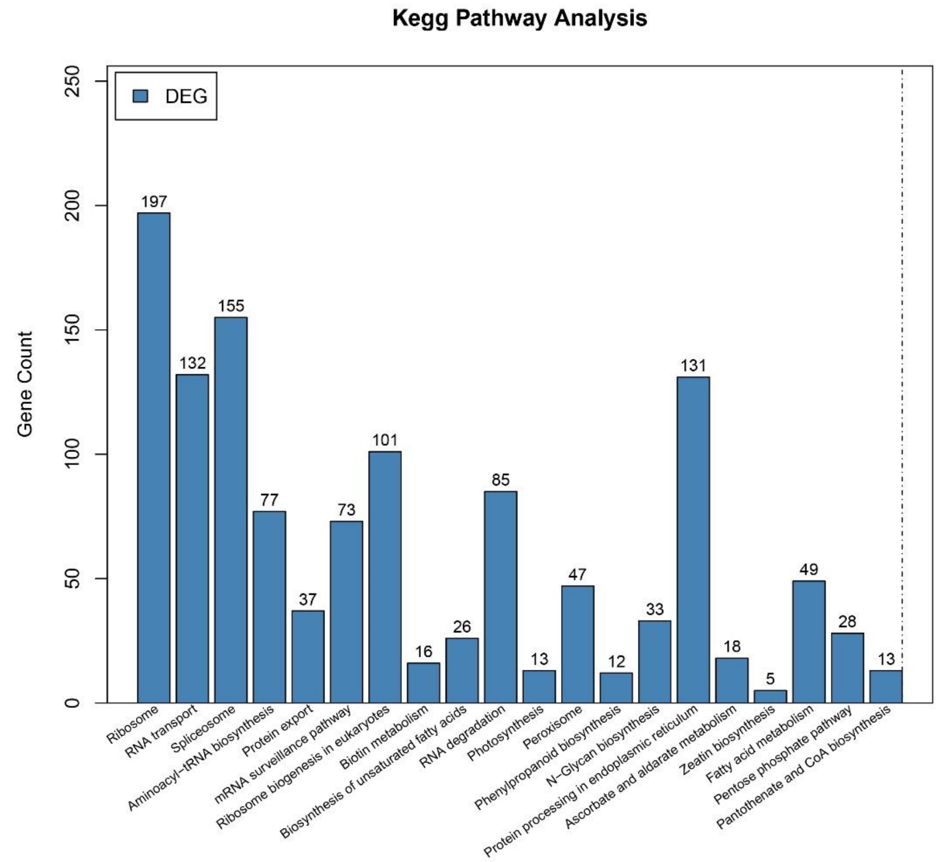

Figure S2. KEGG enrichment analysis in *I. galbana* exposed to MPs compared with the control.

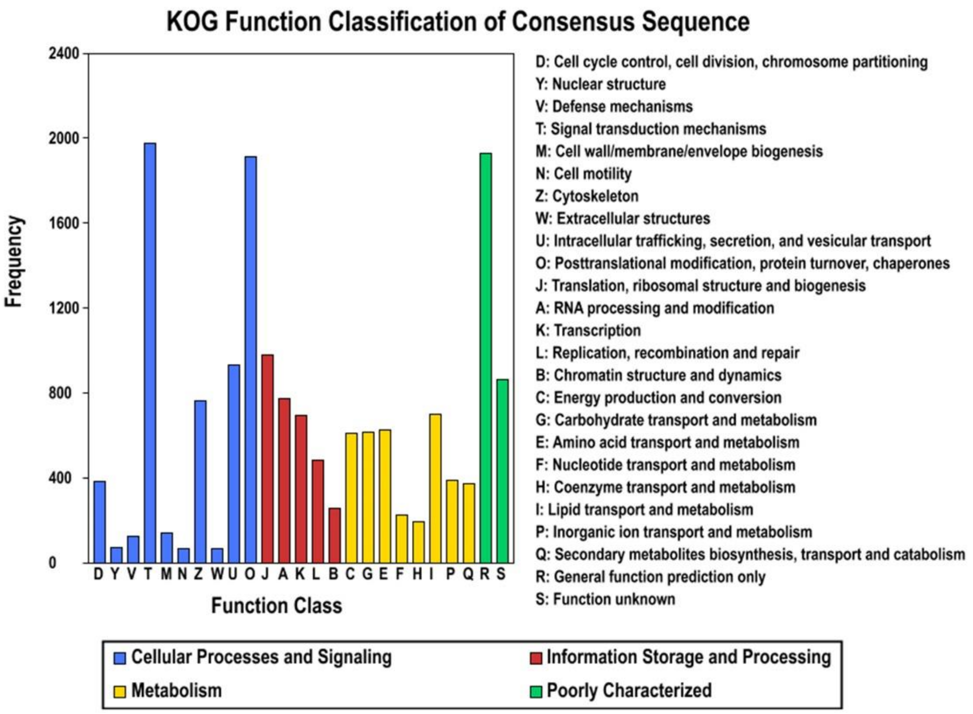

Figure S3. Eukaryotic Orthologous Group (KOG) functional classification. X-axis represents the functional class and the Y-axis represents the number of genes.

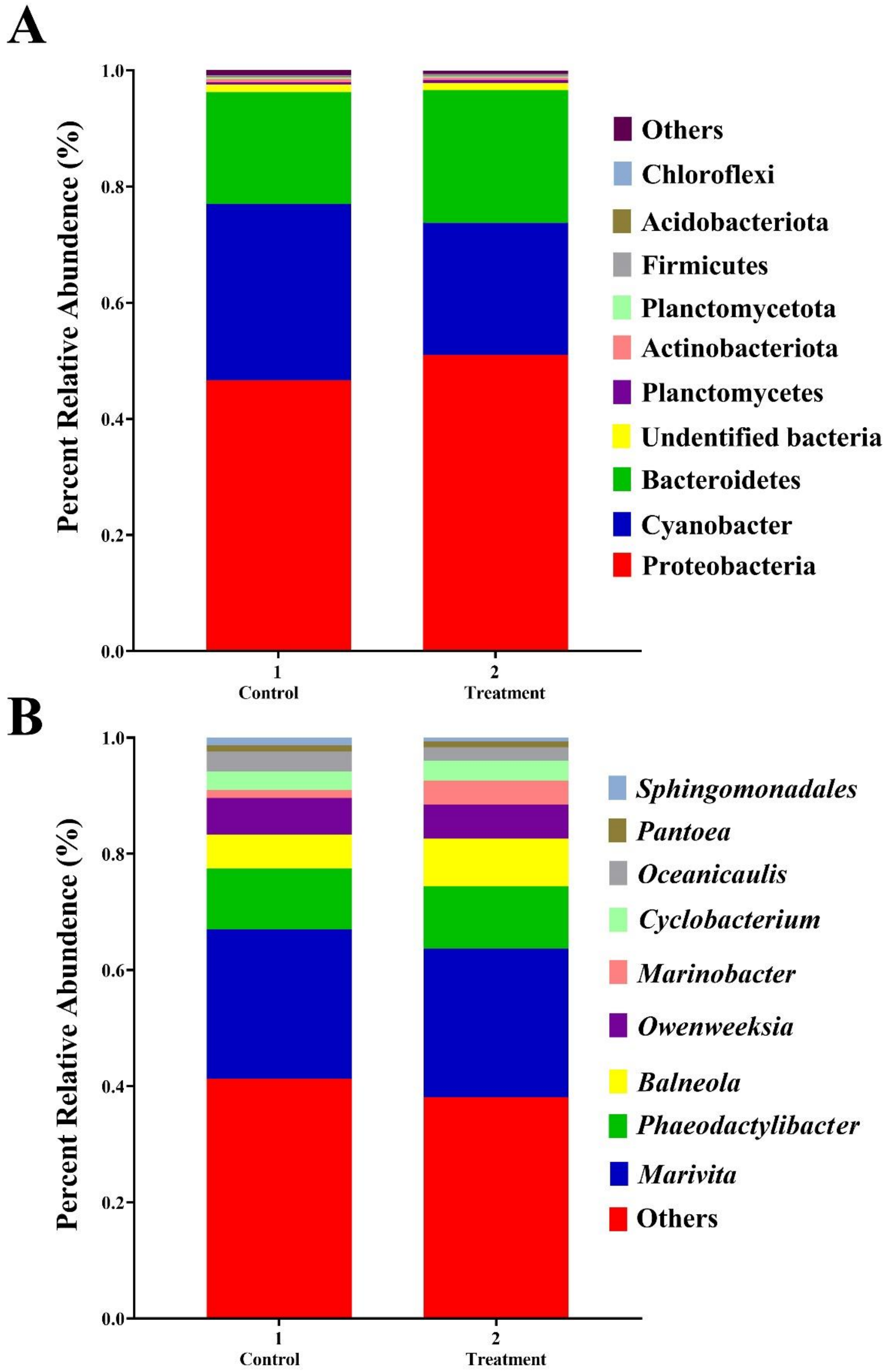

Figure S4. Relative abundance of the dominant bacteria of in different groups at the phylum (A) and genus (B) levels.
